# Biochemical characterization of recombinant *Candida albicans* mannosyltransferases Mnt1, Mnt2 and Mnt5 reveals new functions in *O*- and *N*-mannan biosynthesis

**DOI:** 10.1016/j.bbrc.2012.01.131

**Published:** 2012-03-02

**Authors:** Diana F. Díaz-Jiménez, Héctor M. Mora-Montes, Arturo Hernández-Cervantes, Juan P. Luna-Arias, Neil A.R. Gow, Arturo Flores-Carreón

**Affiliations:** a Departamento de Biología, División de Ciencias Naturales y Exactas, Campus Guanajuato, Universidad de Guanajuato, Noria Alta s/n, Col. Noria Alta, C.P. 36050 Guanajuato, Gto., Mexico; b Departamento de Biología Celular, Cinvestav-IPN, C.P. 07360 Mexico, D.F., Mexico; c School of Medical Sciences, Institute of Medical Sciences, University of Aberdeen, Foresterhill, Aberdeen, AB25 2ZD Scotland, UK

**Keywords:** cpm, counts per minute, ER, endoplasmic reticulum, Mnt, mannosyltransferase, Pmt, protein mannosyl transferase, ORF, Open reading frame, PCR, polymerase chain reaction, TLC, thin layer chromatography, *Candida albicans*, Mannosyltransferases, Glycosylation

## Abstract

The cell surface of *Candida albicans* is enriched with highly glycosylated mannoproteins that are involved in the interaction with host tissues. *N*- and *O*-glycosylation are post-translational modifications that initiate in the endoplasmic reticulum, and finalize in the Golgi. The *KRE2/MNT1* family encode a set of multifunctional mannosyltransferases that participate in *O*-, *N*- and phosphomannosylation. In order to gain insights into the substrate specificities of these enzymes, recombinant forms of Mnt1, Mnt2, and Mnt5 were expressed in *Pichia pastoris* and the enzyme activities characterized. Mnt1 and Mnt2 showed a high specificity for α-methylmannoside and α1,2-mannobiose as acceptor substrates. Notably, they also used *Saccharomyces cerevisiaeO*-mannans as acceptors and generated products with more than three mannose residues, suggesting than Mnt1 and Mnt2 could be the mannosyltransferases adding the fourth and fifth mannose residue to the *O*-mannans in *C. albicans*. Mnt5 only recognized α-methylmannoside as acceptor, suggesting that participates in the addition of the second mannose residues to the *N*-glycan outer chain.

## Highlights

► Mnt5 adds the second residues of lateral branches of the *N*-linked mannan outer chain. ► Mnt1 and Mnt2 participate in the addition of the 4° and 5° residues of *O*-linked mannan. *► Candida albicansO*-linked mannans can be extended further than five mannose residues.

## 1 Introduction

*Candida albicans* is a human opportunistic fungal pathogen that can cause a range of infections from thrush to life-threatening diseases such as systemic candidiasis [1]. Polysaccharides and glycoproteins are the main components of the *C. albicans* cell wall, which is a tough structure that protects the cell from changes in the external environment [2]. Therefore protein glycosylation in fungal pathogens has a particular significance. Mannoproteins are proteins modified with mannose-based oligosaccharides, named mannans, and in *C. albicans* there are three main types of modifications: *O*-mannosylation (oligosaccharides attached to Ser/Thr residues), *N*-mannosylation (glycans attached to Asn residues) and glycosylphosphatidylinositol anchor, where a glycolipid is attached to the C-terminus of polypeptides [3]. The *O*- and *N*-mannosylation pathways have been extensively characterized in *Saccharomyces cerevisiae* and studies in *C. albicans* indicate that these biosynthetic pathways are required for cell wall integrity, dimorphism, adhesion, virulence and recognition by the host innate immune system [1,3–7]. *C. albicans* *N*-mannans are structures composed of a core oligosaccharide (Man_9_GlcNAc_2_) synthesized in the endoplasmic reticulum (ER) and is further modified in the Golgi complex with a α1,6-mannose polymer [6], which is further modified with lateral mannose branches that in *S. cerevisiae* are synthesized by the α1,2-mannosyltransferases Mnn2 and Mnn5, adding the first and second mannose residues to the branches, respectively [8]. The *C. albicans* orthologs to Mnn2 and Mnn5 are predicted to have similar functions during the outer chain formation [9].

*C. albicans* *O*-mannans are linear oligosaccharides composed of up to five α1,2-mannose units [4]. Members of the ER protein *O*-mannosyltransferase family (PMT) add the first mannose residue to nascent proteins. *C. albicans* *PMT* gene family comprises five members *PMT1*, *PMT2*, *PMT4*, *PMT5* and *PMT6* [5]. Disruption of *PMT1* and *PMT6* generates supersensitive mutants to aminoglycoside antibiotics and are less virulent; whereas interruption of *PMT2* and *PMT1*-*PMT4* leads to non-viable mutants, highlighting the importance of *O*-mannosylation for cell viability [5,10,11].

Further modification of *O*-mannans is carried out in the Golgi complex, where mannosyltransferases participate in mannan elongation [12]. Unlike Pmts, the Golgi mannosyltransferases use GDP-mannose as sugar donor [3]. In addition, there are not homologs of these enzymes in mammals, including humans; thus, the study of the catalytic mechanism of these enzymes could potentially unveil new targets for antifungal agents, and get insights in important aspects of fungal cell biology.

*C. albicans KRE2/MNT1* gene family comprises five members belonging to the glycosyl transferases family 15. Bioinformatics analysis of the genes indicate that they encode for type II Golgi α-mannosyltransferases with a high degree of homology (more than 56%) among each other. The biological role of each member of the family has been established: *MNT1* and *MNT2* encode partially redundant α1,2-mannosyltransferases that add the second and third α1,2-mannose residues to the *O*-mannans [4], whereas Mnt3 and Mnt5 have a redundant phosphomannosyltransferase activity, being both involved in the addition of about 50% of the phosphomannose residues presents on the cell wall [13]. *MNT4* and *MNT5* encode for redundant α-mannosyltransferases that catalyze the addition of mannose residues to the *N*-mannan outer chain [13]. The information above described was generated by analyzing the phenotype of null mutant strains lacking specific members of *KRE2*/*MNT1* gene family; however, Mnt1 is the only family member that so far has been biochemically characterized in detail [14]. *C. albicans MNT1* was heterologously expressed in *Pichia pastoris* and recombinant enzyme showed the highest activity when either α-methylmannoside or α1,2-mannobiose was used as acceptor, correlating with the *in vivo* ability of the enzyme to add the second and third mannose residue to *O-*mannan [14]. In order to get more insights about the role of *MNT2* and *MNT5*; we heterologously expressed them in *P. pastoris*, characterized the recombinant enzyme activities, and compared them with recombinant Mnt1 obtained in the same expression system.

## 2 Materials and methods

### 2.1 Organisms and culture media

*C. albicans* NGY152 [15] was used as source of genomic DNA. *P. pastoris* X-33 (Invitrogen) was utilised for gene expression. Cells were propagated in YPD medium [1% (w/v) yeast extract, 2% (w/v) peptone, 2% (w/v) glucose]. YPDS medium [1% (w/v) yeast extract, 2% (w/v) peptone, 2% (w/v) glucose, 1% (w/v) sorbitol, and Zeocin® 100 μg/ml] was used for selection upon cell transformation, whereas MGY medium [1% (w/v) yeast extract, 1.34% (w/v) YNB without amino acids, 2% (w/v) peptone, and 1.64 μM biotin] was used for induction of gene expression. All strains were cultured at 28 °C and shaking at 200 rpm when required.

### 2.2 Construction of the expression plasmids pPMNT1, pPMNT2 and pPMNT5

A PCR product of 1219 bp spanning from nucleotides 91 to 1296 of the *MNT1* open reading frame (ORF) (Genbank ID: X99619) and a product of 1293 bp spanning from nucleotides 106 to 1398 of the *MNT2* ORF (Genbank ID: X89263), were amplified by PCR from genomic DNA using the primer pairs 5′- ATCGATGTGTGGCTATATCCTTACA-3′ and 5′- TCTAGATTAAGCAGTGTACTTTTCCCAAC-3′ for *MNT1* (with the bases to generate *Cla*I and *Xba*I sites underlined), and 5′- GAATTCACCATTACCATCTACGTTC-3′ and 5′- TCTAGATTATTGATATTTTTCCCATTCTTTAG-3′ for *MNT2* (with the bases to generate *Eco*RI and *Xba*I sites underlined). The amplicons were cloned into pCR 2.1-TOPO vector (Invitrogen) and subcloned into *Cla*I-*Xba*I sites of pPICZαC (*MNT1*) and *Eco*RI-*Xba*I sites of pPICZαA (*MNT2*) expression vectors (Invitrogen), generating pP*MNT1* and pP*MNT2* (Supplementary Fig. 1S). Since the CTG codon is recognized in *C. albicans* to incorporate serine instead of leucine into nascent proteins [16], the CTG triplets present in *MNT2* (starting positions at 145 and 373) where changed to TCG using the Quick Change^TM^ II XL site-directed mutagenesis kit (Stratagene). To generate pP*MNT5,* a fragment of the *MNT5* ORF (Genbank ID: AY166654; nucleotides 286 to 1440) was codon optimized for expression in *P. pastoris,* and was synthesized by DNA2.0 (Menlo Park, CA), with *Eco*RI and *Xba*I sites flanking the 1167 bp DNA fragment. Optimized *MNT5* sequence was cloned into the *Eco*RI-*Xba*I restriction sites of the pPICZαA expression vector generating pP*MNT5* (Supplementary Fig. 1S).

### 2.3 Recombinant gene expression in *P. pastoris*

Strain X-33 was transformed by electroporation with 10 μg of *Bst*XI-digested pP*MNT1*, *Sac*I-digested pP*MNT2* or *Sac*I-digested pP*MNT5*. Transformed cells were selected on YPDS-Zeocine medium at 28 °C and positive transformants were grown at the same temperature in 25 ml of MGY medium added with 1% (v/v) glycerol until culture reached *A*_600nm_ = 2. Cells were harvested by low-speed centrifugation, and resuspended in fresh MGY medium added with 1.0% (v/v) methanol. Cells were cultured at 30 °C for 4 days with shaking (200 rpm). Methanol was added to a final concentration of 1.0% (v/v) every 24 h to maintain recombinant gene expression. As controls *P. pastoris* X-33 was transformed with empty vectors pPICZαA and pPICZαC digested with *Sac*I and *Bst*XI, respectively.

### 2.4 Enzymatic activity assays

Mannosyltransferase activity was assayed as described previously [17]. Briefly, assay mixtures contained 50 mM Tris–HCl (pH 7.2), 10 mM MnCl_2_, 0.76 μM GDP-[^14^C] mannose (0.01 μCi; specific activity 262 mCi/mmol), 50 mM α-methylmannoside as acceptor, and 200–300 ng protein. Unless otherwise indicated, standard reactions were performed for 30 min at 30 °C in a volume of 50 μl. The reaction mixtures were passed through 0.4 ml of Dowex 1-X2 anion exchange resin contained in a 1.5 ml Eppendorf tube to remove labeled GDP-mannose. The neutral products were eluted with 1.5 ml of water and radioactivity was determined. Results using supernatants from *P. pastoris* transformed with the empty vector were subtracted from all measured activities. Results were expressed as specific activity and normalized to 1 mg of protein in the culture supernatant.

### 2.5 Protein quantification and analysis

Culture supernatants were analyzed by SDS–polyacrylamide gel electrophoresis (SDS–PAGE) [18] on 12.5% separation gels and stained with Coomassie Brilliant Blue R-250 following standard procedures. Protein was quantified by the Bradford assay using bovine serum albumin as standard [19].

### 2.6 Biochemical analysis of recombinant enzymes

In order to compare substrate specificities, enzyme reactions were carried out in a total volume of 30 μl with 4 mM of each of the following acceptors (all from Sigma Chemical Company): α-methylmannoside, α1,2-D-mannobiose, α1,3-D-mannobiose, α1,6-D-mannobiose, Man_5_(GlcNAc)_2_ and Man_9_(GlcNAc)_2_. Reactions were carried out for 24 h before measuring enzyme activity as described above and specific activity was determined as cpm/mg of protein/h. For metal ion dependence enzyme reactions were carried out for 30 min, using α-methylmannoside as acceptor but the metal cofactor was varied using MnCl_2_, CoCl_2_, ZnSO_4_, MgCl_2_ and CaCl_2_ at 5, 10 and 15 mM concentrations. To test the activity of the mannosyltransferases toward mannooligosaccharides, 10 μl of *O*-linked mannans from *S. cerevisiae* obtained as described below were used as acceptors in standard 50 μl reactions. Reaction mixtures were incubated for 30 min, 2 h or 24 h and the neutral products were concentrated and analyzed by TLC as described afterwards.

### 2.7 Analysis of enzyme products by TLC

Upon enzymatic reaction, the neutral eluted fraction obtained after anion exchange chromatography was concentrated by evaporation and resuspended in 10 μl of deionised water. Then, products were applied to a Silica gel 60 plastic sheet (Merck), and the chromatography was run during 24 h using propanol/butanol/water 12:3:4 as mobile phase. Standards used were glucose, maltose, maltotriose, maltotetraose, maltopentaose, maltohexaose, maltoheptaose, α-metilmannoside, mannose and α1,2-mannobiose (all from Sigma); and were revealed by spraying the plate with a solution of difenilamine/aniline/H_3_PO_4_ and incubating at 100 °C, until spots appearing. Lanes containing radioactive products were divided into one-cm fractions before radioactivity quantification. Reactions with no acceptor substrate were used as controls.

### 2.8 β-elimination of *S. cerevisiae* mannoproteins

Aliquots containing 1 g *S. cerevisiae* mannoproteins (Sigma) were dissolved in 10 ml of NaOH 0.1 M and incubated at room temperature during 18 h with gently shaking. Samples were centrifuged at 2000*g*, supernatants neutralized with HCl, lyophilized and suspended in 3 ml of deionized water. The *O*-linked mannans were kept at −20 °C until use.

### 2.9 Aspergillus satoi a1,2-mannosidase treatment

Enzymatic products derived from incubation of *S. cerevisiae* *O*-mannans with mannosyltransferases were concentrated by evaporation and resuspended with 14 μl of sterile water, 10 μ-units of α1,2-mannosidase (Glyko) and the appropriate buffer were added to a final volume of 20 μl. Enzymatic digestions were carried out at 37 °C during 24 h and then directly applied to a silica 60 plastic sheet. Ascendant chromatography was run and radioactivity was measured as described previously. Reactions without α1,2-mannosidase were used as controls.

## 3 Results

### 3.1 Heterologous expression of *C. albicans* mannosyltransferases in *P. pastoris*

**Fig. 1:**
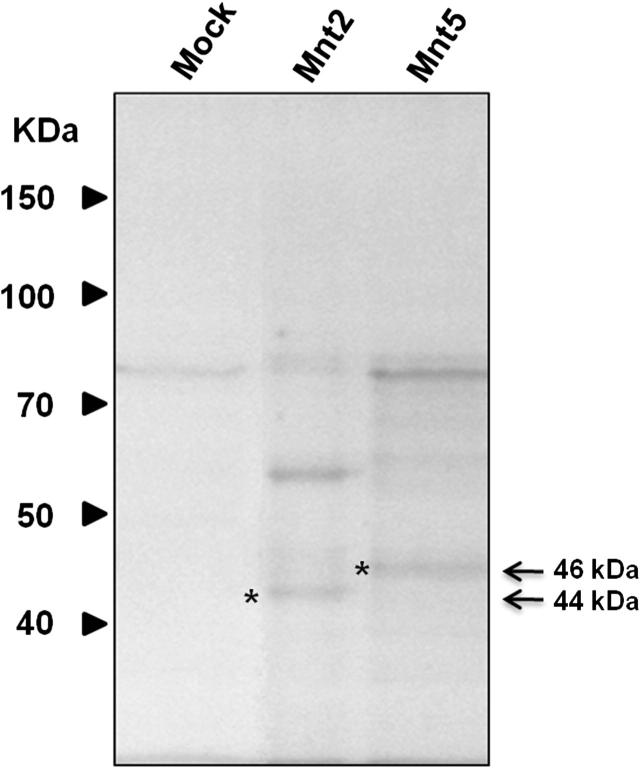
SDS polyacrylamide gel electrophoresis of recombinant Mnt2 and Mnt5 expressed in *P. pastoris*. Proteins were prepared as described in Section 2, and aliquots of 3 μg of protein where analyzed by SDS–PAGE. Samples are: Mock, *P. pastoris* transformed with empty vector; Mnt2, *P. pastoris* transformed with pP*MNT2*; Mnt5, *P. pastoris* pP*MNT5*. The bands corresponding to recombinants proteins are pointed out with asterisks.

To characterize the enzyme activity of *C. albicans* Mnt2 and Mnt5, the *P. pastoris* expression system was used to produce soluble and secreted proteins. The DNA sequences encoding the soluble domain of *MNT2* and *MNT5* were cloned into the pPICZαA expression vector, in frame with the α*-*factor secretion signal sequence, generating pP*MNT2* and pP*MNT5* (Supplementary Fig. 1S). Upon *P. pastoris* transformation with either pP*MNT2* or pP*MNT5*, and induction of gene expression with 1% (v/v) methanol, SDS–PAGE analysis of culture medium revealed two protein bands differentially expressed in cells transformed with pP*MNT2* with molecular weights of 44 and 60 kDa (Fig. 1), whose intensity were dependent on the induction time (data not shown). The expected molecular weight for recombinant Mnt2 was ∼50 kDa, but tandem mass spectrometry identified a 44 kDa protein band as Mnt2 (Supplementary Fig. 2S). For *MNT5* expression, the SDS–PAGE analysis of culture medium from cells transformed with pP*MNT5* and under induction conditions showed an enriched protein differentially expressed with a molecular weight of 46 kDa the predicted molecular weight of the recombinant Mnt5 (Fig. 1).

Mutant analysis predicted that Mnt1 and Mnt2 encode redundant α1,2-mannosyltransferases that participate in *O-*glycosylation [4], therefore we compared the enzymatic properties of these proteins. Recombinant Mnt1 was produced previously in *P. pastoris* GS115 (*his4Δ*) using the vector pHIL-S1 [14]. In order to generate the recombinant enzymes under the same genetic background, the *MNT1* encoding region for the soluble domain was cloned into pPICZαC, generating pP*MNT1* (Supplementary Fig. 1S). This construction was used to transform *P. pastoris* X-33. Recombinant protein was found to be expressed within 24 h of methanol induction (Supplementary Fig. 3S). Optimal induction conditions for the three genes were after two days of incubation with 1% methanol, with yielding 67, 55, and 38 μg/ml of protein for Mnt1, Mnt2 and Mnt5, respectively. The three recombinant enzymes all exhibited mannosyltransferase activity when α-methylmannoside was used as acceptor, with specific activities of 16.1 × 10^5^ ± 2.1 × 10^4^ cpm/mg of protein/min, 10.2 × 10^5^ ± 4.9 × 10^4^ cpm/mg of protein/min, and 2.1 × 10^5^ ± 2.9 × 10^4^ cpm/mg of protein/min, for Mnt1, Mnt2 and Mnt5, respectively.

### 3.2 Properties of recombinant Mnt2 and Mnt5

The pH dependence of enzyme activity was determined in two buffers: PIPES 10 mM between pH 6.4 and 7.2, and Tris–HCl 10 mM between 7.2 and 8.0. Recombinant Mnt1, Mnt2 and Mnt5 showed maximum activity at pH 7.2, when PIPES 10 mM was used as buffer. The optimum activity of recombinant enzymes was found with 10 mM Mn^2+^ (Supplementary Fig. 4S). In addition, activity of Mnt1 and Mnt2, but not Mnt5, was stimulated by Co^2+^ as cofactor, and Mnt1 additionally used Ca^2+^, to a lesser extent (Supplementary Fig. 4S).

Different acceptors were tested as substrates for the enzyme activity of recombinant Mnt2 and Mnt5. Reactions were incubated over 24 h to enable low efficiency reactions to be detected [20]. The reactions were not linear over this period. Mnt2 could utilize α-methylmannoside and α1,2-mannobiose efficiently. The specific activity with α1,2-mannobiose was 6.6 × 10^5^ ± 2.1 × 10^4^ cpm/mg of protein/h, twice than that with α-methylmannoside as acceptor (3.1 × 10^5^ ± 2.5 × 10^4^ cpm/mg of protein/h). This supports the previously predicted *in vivo* function described for Mnt2 [4]. In contrast, no activity was found when mannose, α1,3-mannobiose, α1,6-mannobiose, Man_5_GlcNAc_2_ and Man_9_GlcNAc_2_ were used as acceptors. Mnt5 only showed enzyme activity when α-methylmannoside was used as acceptor sugar, and had a specific activity of 2.2 × 10^5^ ± 2.3 × 10^4^ cpm/mg of protein/h.

### 3.3 Characterization of enzymatic products generated by recombinant Mnt1, Mnt2 and Mnt5

**Fig. 2:**
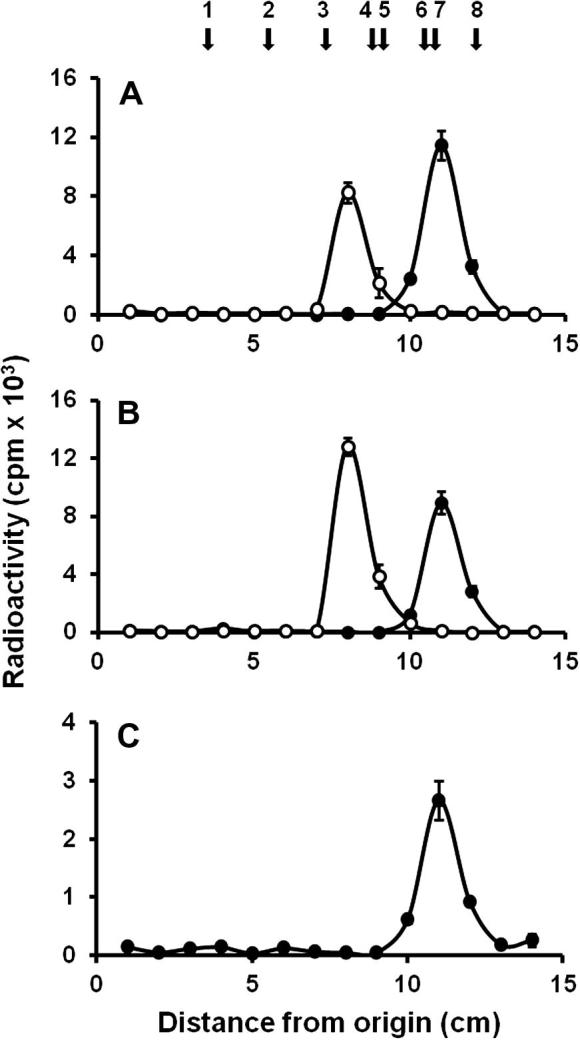
TLC of the products formed by recombinants Mnt1, Mnt2 and Mnt5. Enzymatic products of Mnt1 (A); Mnt2 (B) and Mnt5 (C), using α-methylmannoside (closed circles) or α1,2-mannobiose (open circles) as acceptors. Samples were applied to a silica 60 plastic sheet, separated as described in Section 2, fractions of 1 cm were cut off and radioactivity was measured. Arrows indicate mobility of the following standards (10 μg): 1, maltohexaose; 2, maltopentaose; 3, maltotriose; 4, maltose; 5, α1,2-mannobiose; 6, glucose; 7, mannose; and 8, α-methylmannoside.

The product generated upon enzymatic reactions were then analyzed by TLC. When either α-methylmannoside or α1,2-mannobiose were used as acceptors by Mnt1 or Mnt2, both enzymes were able to add only one mannose unit, generating a disaccharide and trisaccharide, respectively (Fig. 2A and B). Recombinant Mnt5 followed the same trend, adding only one mannose residue to the acceptor α-methylmannoside (Fig. 2C).

**Fig. 3:**
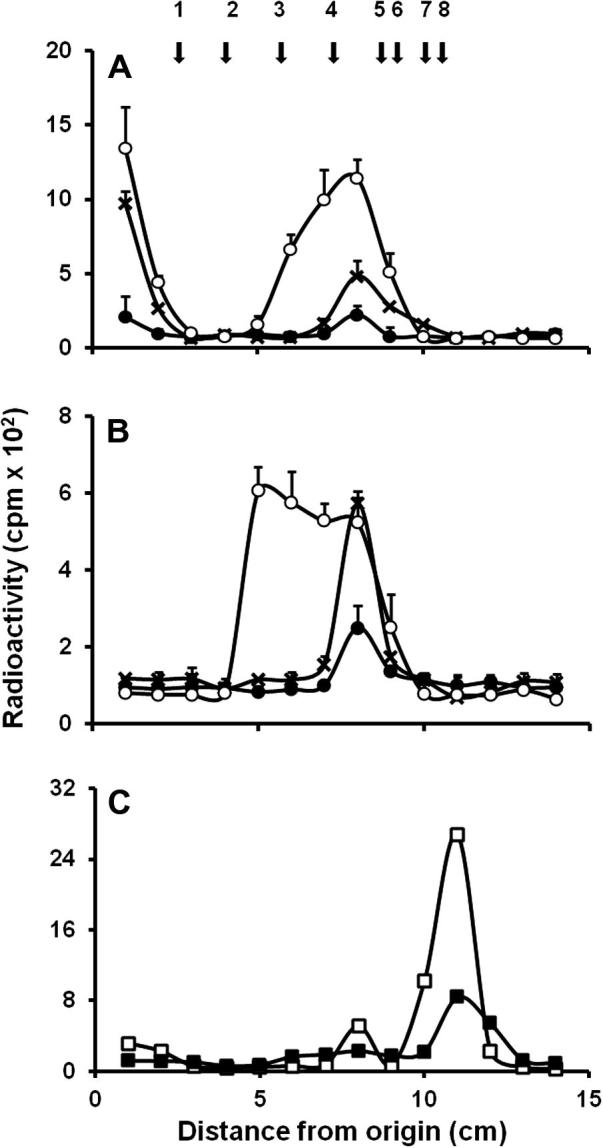
TLC of enzymatic products generated by recombinant Mnt1 and Mnt2 using *S. cerevisiaeO*-mannans as acceptors. *S. cerevisiaeO*-mannans were obtained by β-elimination as described in Materials and Methods, and used as acceptors in mannosyltransferase assays. The enzymatic products of Mnt1 (A) and Mnt2 (B) were obtained after 30 min (closed circles), 2 h (x) and 24 h (open circles) of incubation at 30 °C, and applied to a silica 60 plastic sheet. Products were then separated by TLC, and fractions of 1 cm were analyzed for radioactivity incorporation. (C) the products obtained after long time incubation were then treated with α1,2-mannosidase from *A. satoi* as described in Section 2, the hydrolysis of products of Mnt1 (open squares) and Mnt2 (closed squares) was determined by TLC. Arrows indicate the mobility of the following standards: 1, maltoheptaose; 2, maltohexaose; 3, maltopentaose; 4, maltotriose; 5, maltose; 6, α1,2-mannobiose; 7, glucose and 8, mannose.

In order to assess the ability of recombinant enzymes to use linear mannooligosaccharides as mannose acceptor, *O*-linked mannans from *S. cerevisiae* were prepared and used as acceptors. It has been demonstrated that the β-eliminated *O*-linked mannans have different sizes ranging from one to five mannose units [21]. Recombinant Mnt5 failed to use this glycans as acceptors (data not shown), but recombinant Mnt1 and Mnt2 incorporated significant amount of mannose with these acceptors (Fig. 3). Over short, 2 h incubation times Mnt1 generated two products. One was identified as an oligosaccharide of three mannose units, while the residual radio labeled products remained at the origin of chromatograms. The amount of the products formed was proportional to the incubation time (Fig. 3A). When similar experiments were conducted using recombinant Mnt2, a trisaccharide was identified at short times (Fig. 3B), and a tetrasaccharide appeared after long incubation times (Fig. 3B). In contrast with to the products generated by recombinant Mnt1, recombinant Mnt2 did not synthesize the radioactive material retained at the chromatogram origin (Fig. 3B). Enzymatic products were then digested by α1,2-mannosidase from *A. satoi* (Fig. 3C), supporting the α1,2-mannosyltransferase activity described previously for both enzymes [4]. Linkage of Mnt5 enzymatic product could not be determined.

## 4 Discussion

We used the *P. pastoris* protein expression system to obtain Mnt2, a key enzyme for *O*-glycosylation in *C. albicans*, and Mnt5 a bi-functional enzyme that participates in phosphomannosylation and mannosylation of *N*-linked glycan outer chain. These recombinant enzymes were compared with *C. albicans* Mnt1, whose activity had been characterized previously [4,14]. The biochemical properties of Mnt1 from this study were consistent with those previously reported [14]. Recombinant Mnt2 has a lower molecular weight than the predicted, suggesting the activity of secreted proteases [20,22]. The three enzymes displayed a Mn^2+^-dependent enzyme activity, consistent with the catalytic model site of *S. cerevisiae* Kre2 [20,23]. The highest activity of Mnt2 was achieved using α-methylmannoside and α1,2-mannobiose as acceptors, but not activity was recorded using mannose as acceptor, suggesting that the acceptor must have a substitution in the anomeric carbon, simulating the glycosidic linkage between the first mannose of the *O*-glycans and the glycosylated peptide. Similar observations have been reported for *S. cerevisiae* mannosyltransferases Kre2 and Ktr1 [20]. No activity was found when different disaccharides to α1,2-mannobiose or *N*-mannan core were used as acceptors, suggesting the specificity of these enzymes to recognize the structure of the acceptor.

**Fig. 4:**
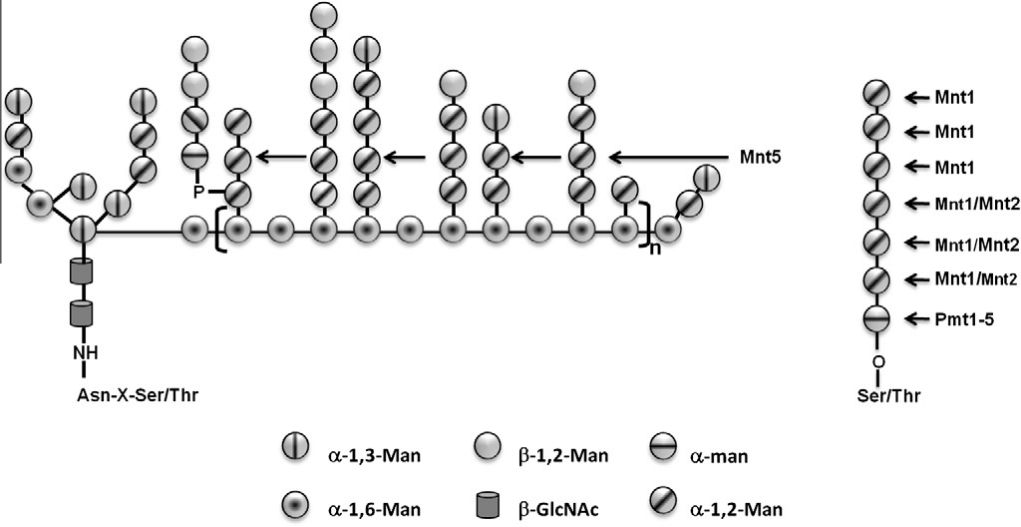
Model of the participation of Mnt1, Mnt2 and Mnt5 in *N*- and *O*-mannan biosynthesis. **Left**, Mnt5 participates in the addition of the second mannose moieties to the lateral branches of the *N*-mannan outer chain. **Right**, Mnt1 and Mnt2 fully extend the *O*-mannan after addition of the first mannose unit by Pmt´s.

Mnt1 preferentially adds the second mannose, whereas Mnt2 adds the third mannose during *O*-glycan elaboration. Here we showed that the enzymes exhibit the same preference for acceptors *in vitro*. In contrast, Mnt5 did not exhibit activity with acceptors other than α-methylmannoside, and would be predicted to yield the product α-methylmannobioside (Fig 3C). Because of the methyl group, this disaccharide does not co-migrate with mannobiose and runs closer to the mannose standard, precluding the identification of glycosidic linkage. It has been suggested that Mnt5 is a bifunctional enzyme that also has phosphomannosyltransferase activity [13], however we failed to demonstrated this activity in the recombinant Mnt5, even when we tried already published methodology [24]. Since the protein showed mannosyltransferase activity, it is likely is properly folded, requires the presence of the positive regulator Mnn4 [25–27]. Since Mnt5 has mannosyltransferase activity only toward α-methylmannoside, we propose that participates adding the second mannose units to the α1,6-mannose backbone (see Fig. 4), along with Mnn5 [9].

Mnt1 and Mnt2 showed ability to transfer mannose units to *S. cerevisiae O*-glycans, generating products with more than three mannose residues; therefore we propose they may also be the mannosyltransferases that add the fourth and fifth moiety to the *C. albicans* *O*-mannans (Fig. 4). This is consistent with other findings that suggest that Mnt enzymes are promiscuous and recognize multiple acceptors [12–14]. *C. albicans* *O*-mannans are linear chains composed of up to five mannose units [4]. The product of Mnt1 that remained at the origin contained more than 7 mannose units, indicating that *C. albicans* *O*-mannans may be larger than seven residues. Accordingly, when *O*-mannans have been analyzed by TLC part of the sample is retained at the origin [4,13,28].
