# Supplementary material for: Biochemical characterization of recombinant Candida albicans mannosyltransferases Mnt1, Mnt2 and Mnt5 reveals new functions in O- and N-mannan biosynthesis
Source: Biochem Biophys Res Commun. 2012 Mar 2;419-248(1):77–82. doi: 10.1016/j.bbrc.2012.01.131 (PMC3480643; doi:10.1016/j.bbrc.2012.01.131)
Supplement: Supplementary data 1 — Supplementary material. [file mmc1.doc]

**Supplementary material**

**Methods**

*Tandem mass spectrometry of the 44 kDa protein*. 10 μg of total protein were loaded in a 12% SDS-polyacrylamide gel. Protein bands were revealed by Coomasie staining and the 44 kDa protein was excised and placed into a small petri dish. Digested band underwent several washes with methanol:acetic acid:water (10:10:80) until Coomasie dye was eliminated, re-hydratation with deionized water was performed when necessary. Band was divided in 1-2 mm^3^ and washed twice with 200 μL of washing solution (methanol-acetic acid-water, 5:1:5, v:v:v). Slices were dehydrated with 200 μL of acetonitrile and polypeptides were reduced with 30 μL of 10 mM DTT, alkylated with 30 μL of 100 mM iodoacetamide for 30 min at RT, and reduced again with 30 μL of 10 mM DTT for 15 min. Gel pieces were dehydrated again as described and incubated with 200 μL of 100 mM ammonium bicarbonate for 10 min at RT, dehydrated with acetonitrile, dried and treated with 35 μL of trypsin (20 ng/μL in 50 mM ammonium bicarbonate, Promega) overnight at 37°C. Next, 30 μL of 100 mM ammonium bicarbonate were added to the gel pieces, incubated for 10 min at 37°C, and centrifuged for 30 s at 8000 x g. Supernantant was recovered and the gel pieces were extracted twice with 30 μL of 100 mM ammonium bicarbonate for 10 min at 37°C and all supernatants were pooled. The volumen was reduced to 15 μL, and filled with 1% acetic acid to 20 μl. Finally, peptides were separated through a Zorbax 200SB C18 reverse phase column (3.5 μm x 75 mm, Agilent Technologies) in an HPLC system (Agilent Technologies 1200 series), using a discontinuos acetonitrile gradient (300 nL/min) (5%, 10%, 20%, 40% and 90% in 1% formic acid in water, 5 min each). Peptides were inmediately analysed in an ESI Q-Trap 3200 MS/MS System (Applied Biosystems) from the Laboratorios Centrales del Cinvestav-IPN in Mexico City. The identification of peptides was performed with the ProteinPilot software version 2.0.1 using the UniProtKB/SwissProt and TrEMBL databases.

**Figures legends**

**Fig. 1S**. **Map of expression plasmids pP*MNT1* pP*MNT2* and pP*MNT5***. *5´AOX1*, *P. pastoris* *AOX1* gene promoter; *α-factor*, secretion signal of the *S. cerevisiae* mating α factor; *MNT1* soluble domain (A), *MNT2* soluble domain (B) and *MNT5* soluble domain (C); *AOX(TT),* AOX transcription terminator; PTEF1, Promoter of the *S. cerevisiae* transcription elongation factor 1 gene; PEM7, synthetic prokaryotic promoter; *Zeocin*, zeocin resistance gene; CYC1, transcription terminator of the *S. cerevisiae* *CYC*1 gene; pCU ori, origin of replication.

**Fig. 2S. Molecular mass profile of the 44 kDa protein fragments**. The 44 kDa protein was excised from the 12% polyacrylamide gel and underwent to digestion with trypsin, the fragments generated were analysed by tandem mass spectrometry. The mass spectrums show the sequence of the peptides identified. Peptides are representative of *C. albicans* Mnt2 sequence.

**Fig. 3S. SDS polyacrylamide gel electrophoresis of recombinant Mnt1 expressed in *P. pastoris*.** Protein induction in medium containing methanol 1%, the culture medium was separated from cell package, and aliquots of 3 mg of protein at 1-5 days post-induction, where analysed by SDS-PAGE. Samples are: Mnt1, *P. pastoris transformed* with pP*MNT1* (lanes 1-5) after 1, 2, 3, 4 and 5 days of methanol induction; Mock, *P. pastoris* transformed with empty vector after 5 days of methanol induction.

**Fig. 4S**. Metal ion dependence of recombinants Mnt1, Mnt2 and Mnt5. The concentration of different divalent cations was varied from 0 to 15 mM in standard mannosyltransferase assays, containing recombinant Mnt1 (A), Mnt2 (B) or Mnt5 (C), as described in Materials and Methods. Cations tested were: Mn^2+^ ■, Co^2+^ ▲, Ca^2+^ **+**, Mg^2+^ ○ and Zn^2+^ ♦. Specific activity = cpm/mg of protein/min.

**Reference**

Kinter M, and Sherman NE. 2000. Protein sequencing and identification using tandem mass spectrometry. Kinter M and Sherman NE Editors. Wiley-Interscience, Inc. pp. 147-165. New York, U.S.A.

**Figure 1S**

**
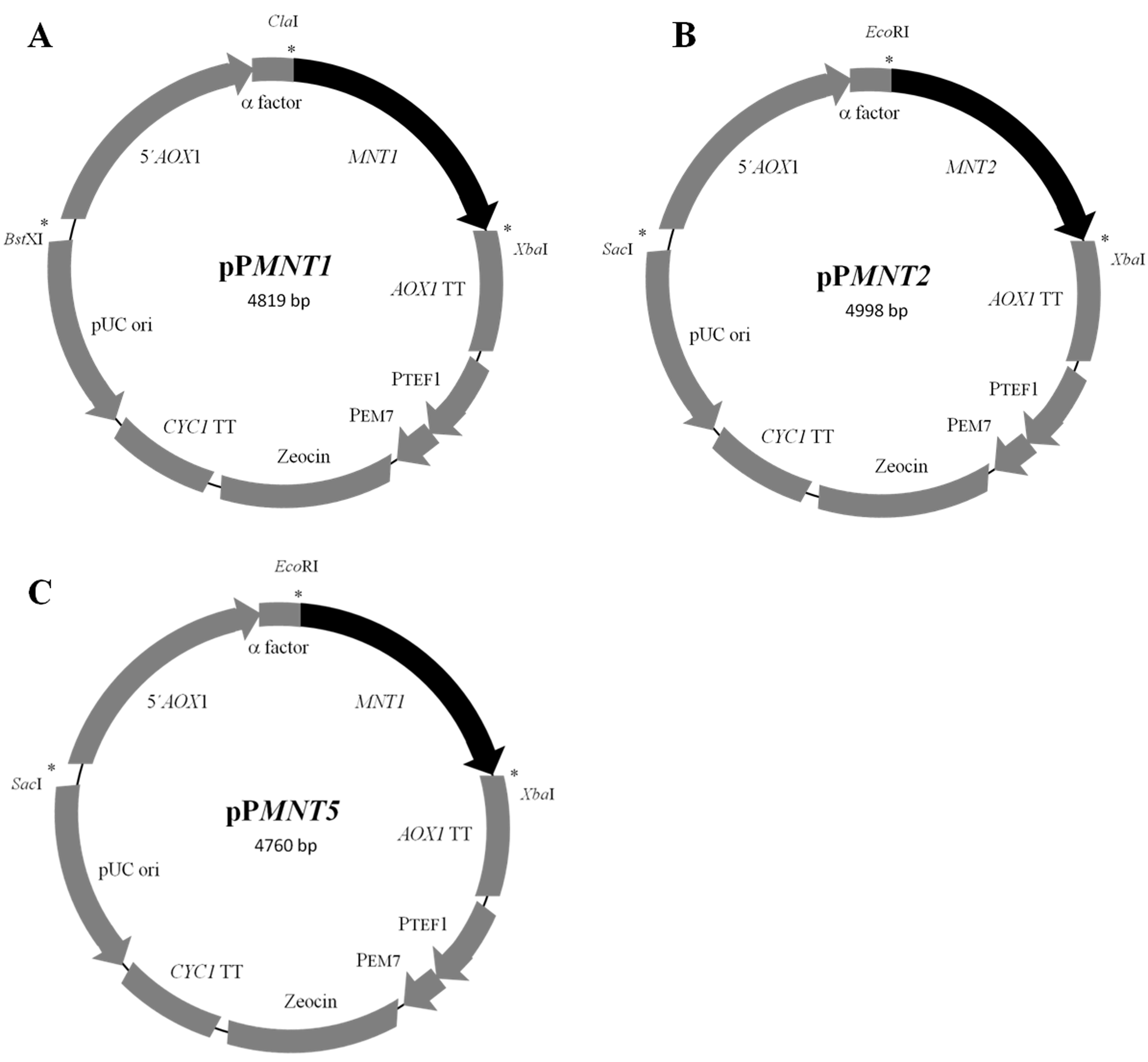
**

**Figure 2S**

**
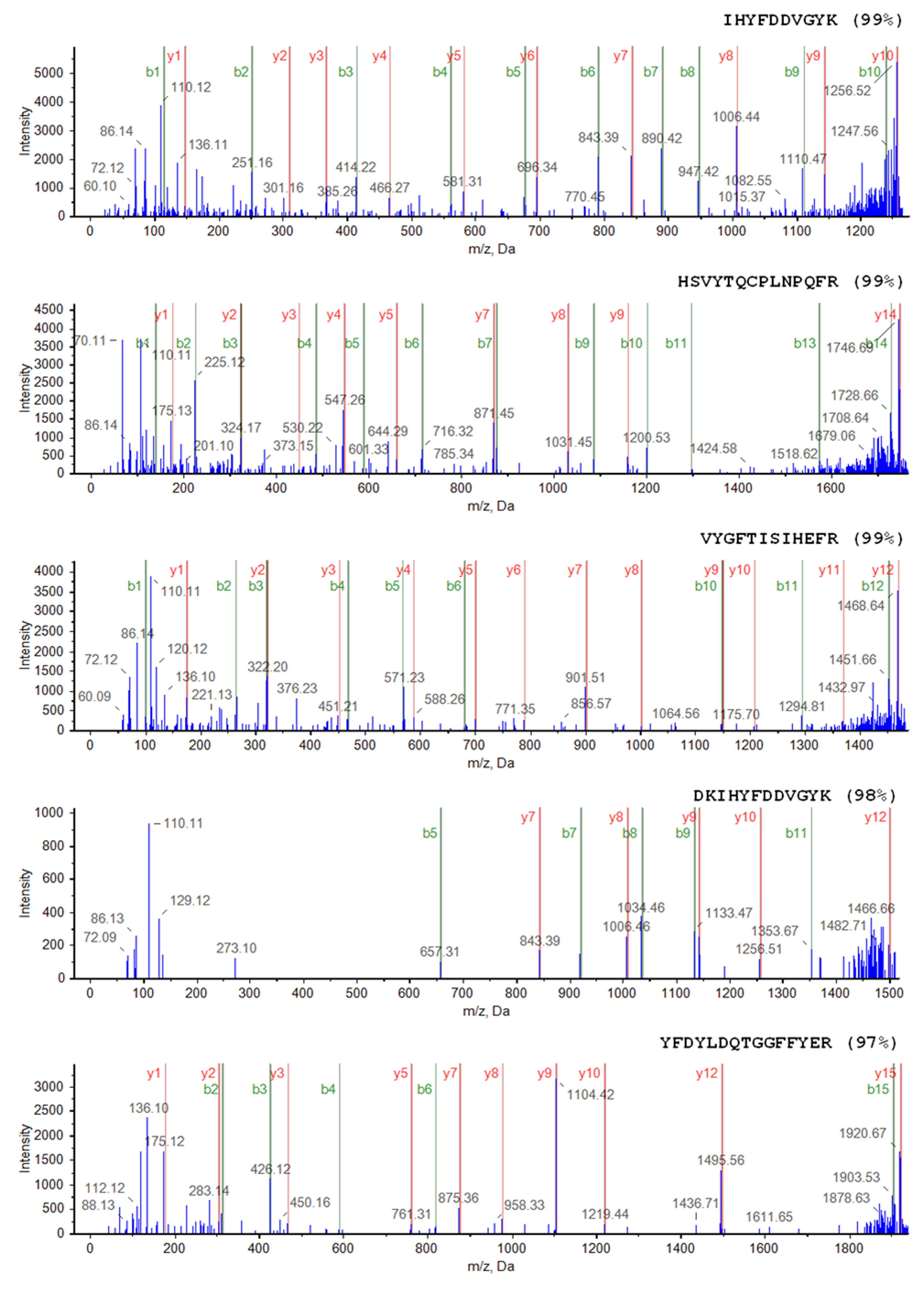
**

**Figure 3S**

**
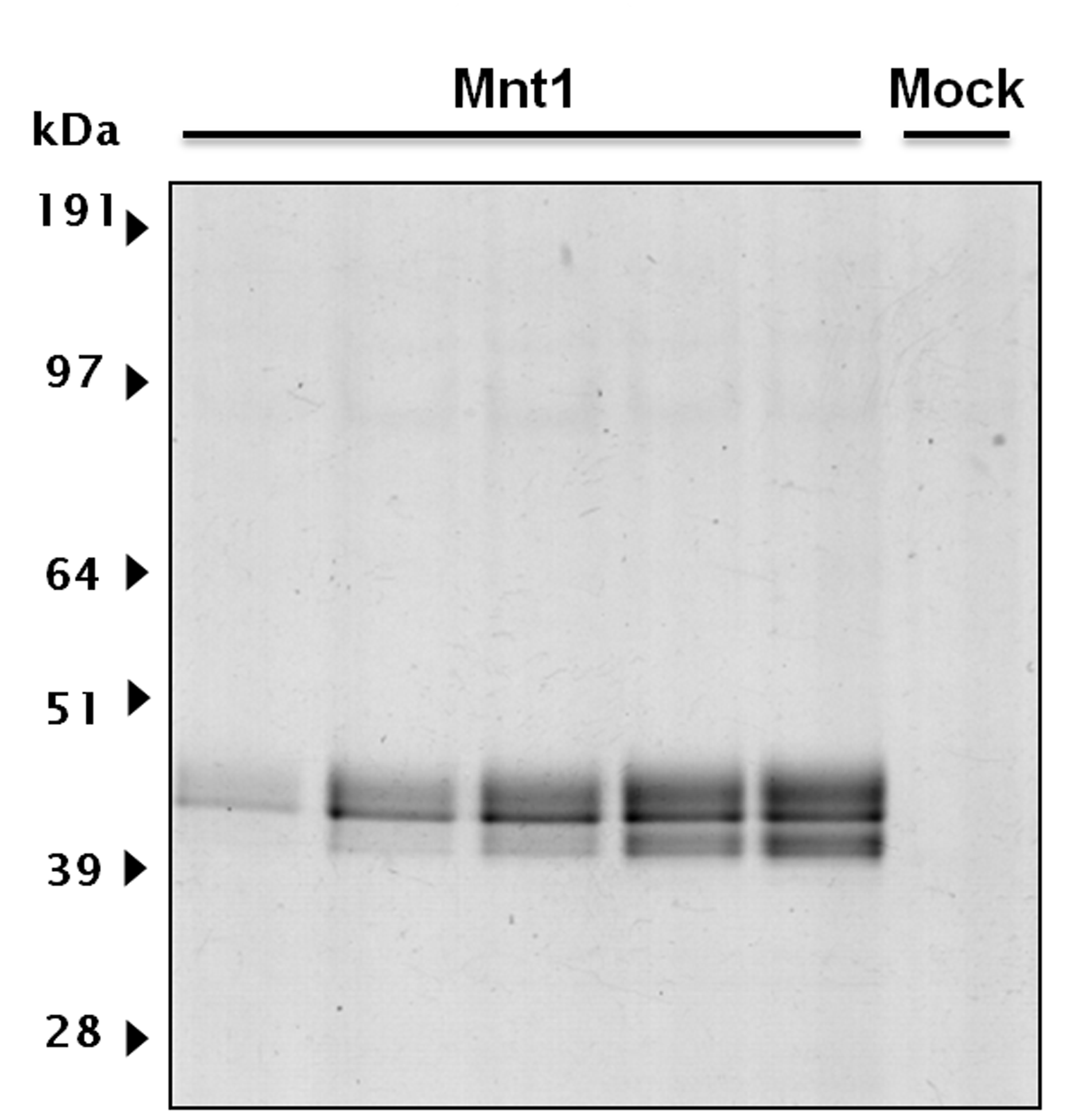
**

**Figure 4S**

**
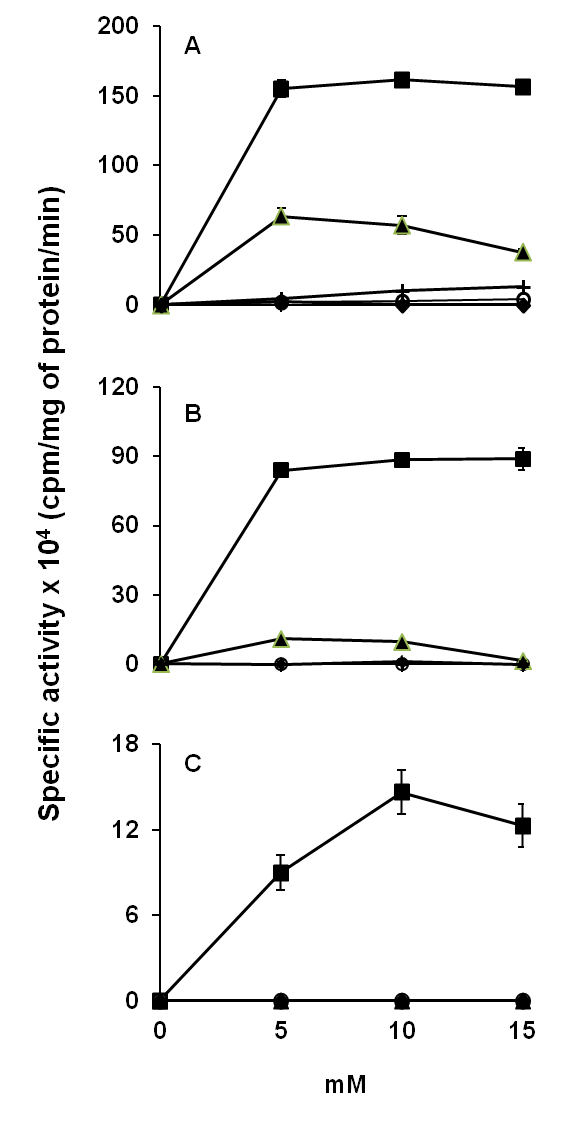
**
